# Oral Supplementation of *Bacillus subtilis* Attenuated Alveolar Bone Loss in a Mouse Model of Ligature-Induced Periodontitis

**DOI:** 10.3390/dj14080494

**Published:** 2026-08-07

**Authors:** Yixuan Zhang, Naoki Toyama, Mohammad Nurhamim, Momoko Nakahara, Daiki Fukuhara, Takayuki Maruyama, Daisuke Ekuni

**Affiliations:** 1 Department of Preventive Dentistry, Graduate School of Medicine Dentistry and Pharmaceutical Sciences, Okayama University, 2-5-1 Shikata-cho, Kita-ku, Okayama 700-8558, Japan; py466nau@s.okayama-u.ac.jp (Y.Z.); dr.nurhamim@gmail.com (M.N.); 2 Dental School, Okayama University, 2-5-1 Shikata-cho, Kita-ku, Okayama 700-8558, Japan; pu171qxi@s.okayama-u.ac.jp (N.T.); de20041@s.okayama-u.ac.jp (D.F.); 3 Department of Preventive Dentistry, Academic Field of Medicine, Dentistry and Pharmaceutical Sciences, Okayama University, 2-5-1 Shikata-cho, Kita-ku, Okayama 700-8558, Japan; pric37ll@s.okayama-u.ac.jp (M.N.); t-maru@md.okayama-u.ac.jp (T.M.)

**Keywords:** *Bacillus subtilis*, periodontitis, alveolar bone loss, gut microbiota, intestinal morphology, vitamin D, mouse model

## Abstract

**Background/Objectives:** This study aimed to investigate the effects of oral *Bacillus subtilis* (BS) on alveolar bone loss, intestinal morphology, and gut microbiota in a mouse model of ligature-induced periodontitis. **Methods:** A total of 24 male C57BL/6J mice (6 weeks old) were allocated to control, periodontitis (P), BS, and BS+P groups. Periodontitis was induced by bilateral ligation of maxillary second molars, and BS was orally administered for 18 consecutive days. Gut microbiota composition was analyzed by 16S rDNA sequencing, alveolar bone loss and gut morphology were evaluated using ImageJ version 1.54g, and ELISA-detectable serum vitamin D metabolite concentrations were measured using an enzyme-linked immunosorbent assay. **Results:** Compared with the P group, the BS+P group showed reduced bone loss. Serum vitamin D metabolite concentrations, small-intestine villus height, and villus height-to-crypt depth ratios were higher in the BS+P group. In gut microbiota, alpha diversity differed significantly among groups based on the Shannon index. Bray–Curtis-based beta diversity differed significantly among groups. **Conclusions:** Oral supplementation of BS attenuated the progression of ligature-induced periodontitis in a mouse model, and changes in gut microbiota may be associated with this effect.

## 1. Introduction

Periodontitis is a chronic inflammatory disease resulting in the destruction of the gingiva, periodontal ligament, cementum, and alveolar bone, potentially leading to tooth loss [1]. It is primarily driven by dysbiosis of the oral microbiome and host immune responses and induces systemic implications that extend beyond the oral cavity, such as diabetes, pregnancy complications, and cardiovascular disease [2,3].

Microbial diversity is commonly assessed as alpha diversity within samples and beta diversity between samples. In severe periodontitis, fecal alpha diversity may remain unchanged despite differences in beta diversity and microbial composition [4], which may further promote the progression of various systemic diseases. Some salivary microbiota from patients with periodontitis can translocate to the gut and induce dysbiosis. Periodontitis-related salivary microbiota has been shown to induce gut microbial dysbiosis, leading to the exacerbation of systemic inflammation and aggravation of Alzheimer’s disease [5]. Additionally, periodontitis has been reported to influence the progression of diseases such as hypertension [6], chronic liver disease [7], and prediabetes [8] through the induction of intestinal dysbiosis. On the other hand, gut microbiota may influence the progression of periodontitis. Some studies have reported the important role of gut microbiota in periodontitis via the gut–alveolar bone axis [9]. In addition, some specific gut microorganisms have shown potential effects on periodontal disease. For instance, *Eubacterium xylanophilum* and *Lachnoclostridium* were associated with reduced gum bleeding; *Anaerotruncus*, *Eisenbergiella*, and *Phascolarctobacterium* were linked to a lower risk of periodontal disease; and *Fusicatenibacter* was associated with an increased risk of periodontal disease [10]. However, whether the progression of periodontitis can be modified by regulating the gut microbiota remains unknown.

Strategies for modulating the gut microbiota include probiotics, prebiotics, postbiotics, antibiotics, and fecal microbiota transplantation. Among these strategies, probiotics such as *Lactobacillus* spp., *Bifidobacterium* spp., *Akkermansia* spp., and *Faecalibacterium* spp. are part of widely used gut microbiota management strategies for improving host health [11]. Probiotics can help improve gastrointestinal health and gut barrier function and affect both the mucosal and systemic immune systems [12,13,14]. In recent years, probiotics have also been explored as an adjunct therapy for the treatment of oral diseases, including dental caries and periodontitis [15]. Some clinical trials have demonstrated that *Lactobacillus* spp. and *Bifidobacterium* spp. show efficacy in improving gingival and periodontal health [16,17,18]. However, the potential role of other probiotics in treating periodontitis remains unclear, as do the mechanisms underlying their potential benefit for periodontal health.

*Bacillus subtilis* (BS) is an emerging probiotic that has been widely studied for its immunomodulatory properties as well as its ability to enhance gut barrier integrity and regulate intestinal flora [19,20]. BS has been demonstrated to improve body growth performance [21], reduce gastrointestinal symptoms [22], and improve blood lipids and endothelial function [23]. Furthermore, pretreatment of BS and *Bacillus licheniformis* reduced alveolar bone loss in experimental periodontitis in rats and enhanced the effects of periodontal treatment [24]. However, the effects of BS administration after periodontitis induction on alveolar bone loss and gut microbiota remain insufficiently characterized. Thus, we focused on BS because BS administration may be a potential new treatment for periodontitis.

Therefore, this study aimed to investigate the effects of oral BS on alveolar bone loss, intestinal morphology, and gut microbiota in a mouse model of ligature-induced periodontitis.

## 2. Materials and Methods

### 2.1. Experimental Design and Animal Model

All specific pathogen-free male C57BL/6J mice (6 weeks old) were purchased from Japan SLC, Inc. (Shizuoka, Japan). All mice in healthy condition were included in this study. The exclusion criterion was reaching a humane endpoint, as defined below. All mice were housed at a controlled temperature (23–25 °C) and subjected to a regular 12 h light/12 h dark cycle. A standard MF diet (Oriental Yeast Co., Ltd., Tokyo, Japan) and water were provided ad libitum.

**Figure 1:**
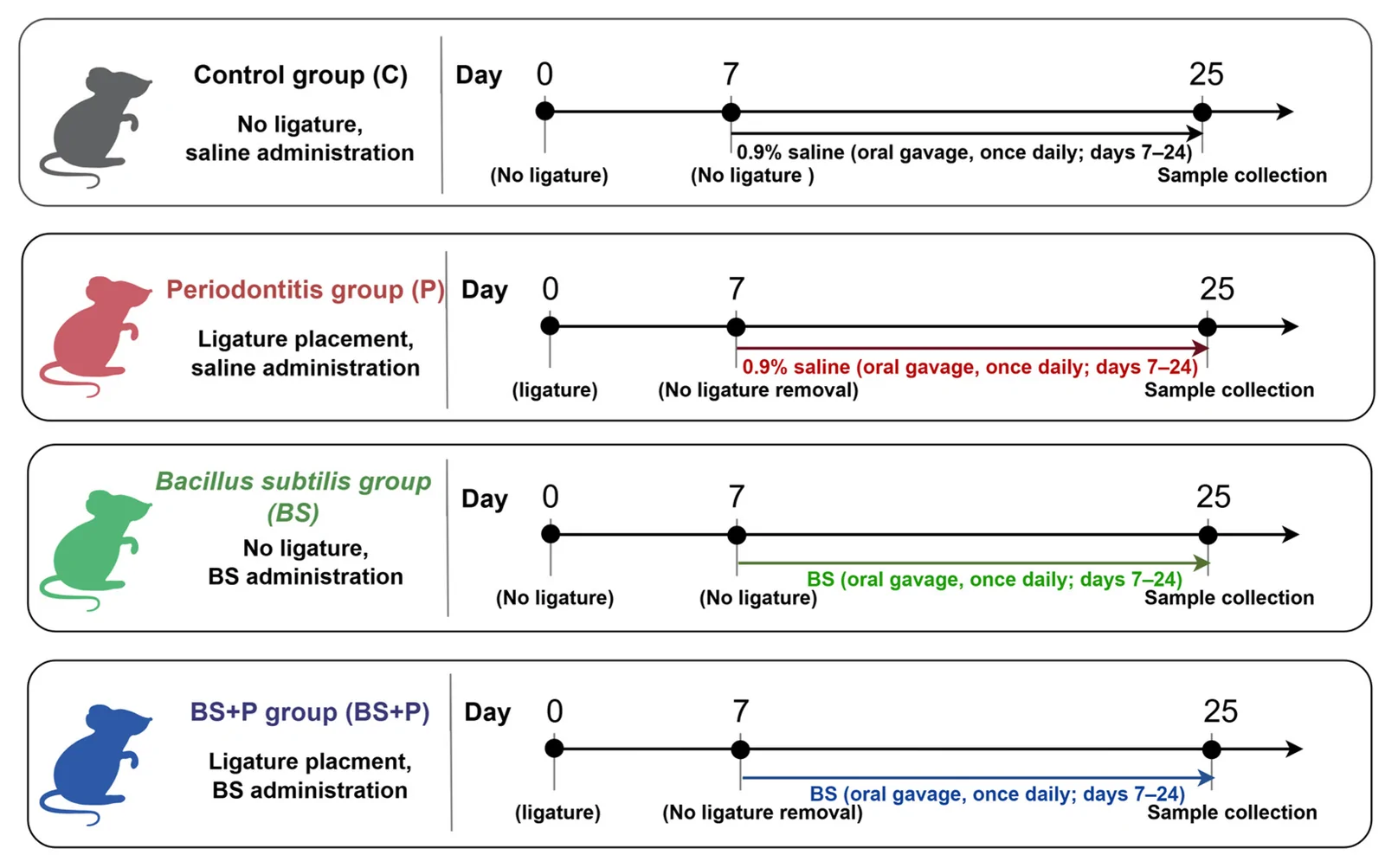
Experimental design of oral *Bacillus subtilis* supplementation in mice with ligature-induced periodontitis. Mice were allocated to the control (C), periodontitis (P), *Bacillus subtilis* (BS), and *Bacillus subtilis* plus periodontitis (BS+P) groups. Ligatures were added on day 0 and maintained until sample collection on day 25 in the P and BS+P groups. From day 7 to day 24, mice received either 0.9% saline or BS by oral gavage once daily. Tissue and fecal samples were collected on day 25. The figure was created by Figdraw.

The mouse ligature-induced periodontitis model was used because it enables concurrent assessment of periodontal destruction and systemic responses in vivo [25,26]. These integrated outcomes cannot be adequately reproduced using in vitro cell cultures or isolated tissues. A sample size (*n* = 6/group) was calculated based on a previous mouse ligature-induced periodontitis study (effect size; 0.25, alpha; 0.05, power; 0.80) following the reduction principle of the 3Rs. Multiple outcomes were obtained from each animal to avoid the use of separate animal cohorts. A total of 24 male C57BL/6J mice were allocated to four groups (*n* = 6 per group) by a quasi-randomization sequence without a random number table (Figure 1).

Control group (C): Mice received 0.5 mL of normal saline (Otsuka Pharmaceutical, Tokushima, Japan) via oral gavage once daily for 18 days, starting from day 7. No ligature was added. This group served as a negative control for baseline physiological and histological comparisons.

Periodontitis group (P): Periodontitis was induced on day 0 by placing a 5-0 silk ligature (IA05SB, Nesco Suture^®^; Alfresa Pharma Corporation, Osaka, Japan) around the bilateral maxillary second molars under general anesthesia induced by intraperitoneal administration of medetomidine (0.3 mg/kg, Domitor^®^; Orion Corporation, Espoo, Finland), midazolam (4 mg/kg; Sandoz, Tokyo, Japan), and butorphanol (5 mg/kg; Meiji Animal Health, Kumamoto, Japan). After a 7-day induction period, mice received 0.5 mL of 0.9% saline by oral gavage once daily for 18 consecutive days (days 7–24). The ligatures were retained without removal until sample collection on day 25 [27,28].

BS group: Mice were administered BS (1.0 × 10^9^ colony-forming units (CFU)/mL, 0.5 mL) via oral gavage once daily from day 7 to day 24, without any ligature placement [29]. BS powder was obtained from TOA Biopharma Co., Ltd. (Tokyo, Japan).

BS+P group (BS+P): Periodontitis was induced as described for the P group. Beginning on day 7, mice received 0.5 mL of a BS suspension (1.0 × 10^9^ CFU/mL) by oral gavage once daily for 18 consecutive days (days 7–24). The ligatures were retained without removal until sample collection on day 25.

Investigators performing the experimental interventions were aware of group allocation because the ligatures were visible, whereas investigators performing outcome assessment were blinded to group allocation. Throughout the 25-day experiment, all mice were monitored daily for changes in appearance, posture, activity, feeding behavior, and pain-related facial expressions, assessed qualitatively with reference to the Mouse Grimace Scale described by Langford et al. [30]. Ligature placement was not surgery and did not induce pain after the subjects woke up. Therefore, routine daily postoperative analgesia was not considered necessary in the approved protocol. Body weight was measured on days 0, 7, and 25. Humane endpoints were ≥20% weight loss from the previous measurement or intolerable pain or distress; affected animals would be euthanized by isoflurane overdose followed by exsanguination. No animals showed clinical signs requiring intervention, reached these endpoints, or were excluded. No animals died before study completion, and no ligatures were lost before the endpoint. No animals or specimens were excluded from the primary analyses. Sample C2 was retained in the primary microbiome analysis and was excluded only in the sensitivity analysis because of its low sequencing depth.

### 2.2. Sample Collection and Processing

Fecal samples were collected individually from each mouse on day 25 to characterize endpoint fecal microbial community composition. Approximately 0.1–0.2 g of freshly excreted feces was collected from each mouse and stored at −80 °C until further processing [31].

Terminal blood collection was performed via the heart under deep anesthesia, immediately followed by euthanasia. Approximately 0.3–0.5 mL of blood was collected per animal and centrifuged at 1500× *g* for 15 min to separate the serum. The resulting serum was stored at −80 °C until analysis, including the measurement of serum vitamin D concentrations.

Following euthanasia on day 25, maxillary bones, including the second molars, were dissected from each mouse. The samples were stored to measure alveolar bone loss.

Intestinal tissues were collected for histopathological analysis, focusing on intestinal villus height (IVH), intestinal crypt depth (ICD), and the villus-to-crypt ratio.

### 2.3. Measurement of ELISA-Detectable Serum Vitamin D Metabolite Concentrations

Serum vitamin D metabolites were measured using a competitive Vitamin D ELISA kit (Item No. 501050; Cayman Chemical, Ann Arbor, MI, USA) according to the manufacturer’s instructions [32]. An eight-point standard curve (0.19–25 ng/mL) was generated using the 25-hydroxyvitamin D_3_ [25(OH) vitamin D_3_] standard. Samples were analyzed in duplicate at 1:10 dilution. Quantitative results were calculated from the 1:10 dilution using a plate-specific logit–log standard curve and corrected for the dilution factor. Absorbance was measured at 420 nm using a microplate reader (SH-1000Lab; Corona Electric Co., Ltd., Ibaraki, Japan). The total amount of 25-OH and/or 1α,25-(OH)_2_ vitamin D_3_ level was measured.

### 2.4. Measurement of Alveolar Bone Loss

Maxillary bone samples were stained with 1% methylene blue (Sigma, St. Louis, MO, USA) to enhance the visualization of the alveolar bone structures [33]. Images were captured using a digital camera (Nikon Instruments, Tokyo, Japan) and analyzed using ImageJ software version 1.54g (National Institutes of Health, Bethesda, MD, USA). Periodontitis was induced bilaterally; however, alveolar bone loss was assessed on the left maxillary side of each mouse. Three linear distances from the cementoenamel junction (CEJ) to the alveolar bone crest (ABC) were measured at predetermined buccal sites of the second molar and averaged to obtain one mouse-level CEJ–ABC distance [33]. CEJ–ABC distances were also normalized to the mean value of the C group, which was defined as 100%, and expressed as control-normalized relative CEJ–ABC distances. Statistical analyses were performed using the original values in millimeters.

### 2.5. Gut Microbiota Analysis

Total bacterial DNA was extracted from fecal samples collected on day 25 using a commercially available DNA extraction kit (Genome Lead Co., Ltd., Kagawa, Japan). To characterize the microbial composition of the gut, 16S rDNA sequencing was conducted, focusing on the V3–V4 regions. Amplicon libraries were constructed by two-step PCR using KAPA HiFi HotStart polymerase, purified with AMPure XP beads, dual-indexed, quantified, and normalized.

Paired-end sequencing was performed on an Illumina MiSeq platform using the MiSeq Reagent Kit v3 600-cycle chemistry (Illumina, San Diego, CA, USA), generating 301 bp paired-end reads. The final library concentration was 12 pM, and 20% PhiX was added to the sequencing run. Primary bioinformatic processing was performed using QIIME 2 [34]. Low-quality, PhiX, and chimeric sequences were removed using DADA2, and an ASV feature table was generated.

Before downstream analysis, the ASV table was filtered using a prevalence-based criterion. ASVs were retained in the dataset if they were present in at least 4 of the 24 samples. Downstream microbiota analyses were performed in MicrobiomeAnalyst 2.0 using identical filtering settings across all comparisons [35]. The low-count filter was set to 0 with a 10% prevalence threshold, and the low-variance filter was set to 0%. Alpha diversity was assessed using the Shannon and Simpson indices, and beta diversity was evaluated using Bray–Curtis dissimilarity with principal coordinate analysis, with group-level differences tested using PERMANOVA. Discriminatory taxa were explored using LEfSe, and taxon-level patterns were visualized by heat tree analysis. Rarefaction curves were examined to assess sequencing depth and saturation across samples.

The raw sequencing reads generated from the 16S rRNA gene amplicon sequencing analysis were deposited in the NCBI Sequence Read Archive under BioProject accession number PRJNA1480160. The corresponding sample names, BioSample accession numbers, and SRA Run accession numbers are provided in Supplementary Table S1.

### 2.6. Histopathological Analyses

For histopathological analyses, intestinal tissues were fixed in 4% paraformaldehyde for 24 h. After fixation, the samples were dehydrated through a graded ethanol series (70%, 80%, 90%, and 100%) and embedded in paraffin. Tissue sections were cut at a thickness of 4 μm using a microtome (Yamato Kohki Industry, Saitama, Japan) and subsequently stained with hematoxylin and eosin (H&E; FUJIFILM Wako Pure Chemical Corporation, Osaka, Japan) for histological analysis. Tissue slides were examined under a microscope (BZ-X810; Keyence, Itasca, IL, USA) and processed for evaluation of IVH, ICD, and the villus-to-crypt ratio (V/C), which are key indicators of the effective absorption area and can be affected by probiotics [36].

### 2.7. Measurement of Body Weight

Body weight was measured on days 0, 7, and 25 using a calibrated electronic balance (Model CJ-2200; Shinko Denshi Co., Ltd., Tokyo, Japan). Measurements were performed at approximately the same time of day.

### 2.8. Statistical Analysis

Statistical analyses were performed with GraphPad Prism version 10.1.2 (GraphPad Software, Boston, MA, USA) and MicrobiomeAnalyst 2.0. Parametric data are presented as mean ± standard deviation (SD), whereas gut microbiome data are presented as the median with interquartile range (IQR). Body weight, CEJ–ABC distance, ELISA-detectable serum vitamin D metabolite concentrations, and intestinal morphological parameters were analyzed using all animals according to their original group allocation (*n* = 6 per group). For intestinal histomorphometric outcomes, measurements obtained within each mouse were averaged before statistical analysis, and the mouse was treated as the experimental unit.

Normality and variance homogeneity were assessed using the Shapiro–Wilk and Brown–Forsythe tests, respectively. Normality was not rejected for the parametric outcomes. Variance homogeneity was not rejected for CEJ–ABC distance, small-intestine villus height, or small-intestine crypt depth, whereas heterogeneity was detected for the villus-to-crypt ratio and serum vitamin D metabolite concentrations.

CEJ–ABC distance and small-intestine villus height were analyzed using one-way ANOVA followed by Tukey’s test. Serum vitamin D metabolite concentrations, small-intestine crypt depth, and the villus-to-crypt ratio were analyzed using Welch’s ANOVA followed by Dunnett’s T3 test. Large-intestine crypt depth was analyzed using the Kruskal–Wallis test followed by FDR-corrected Mann–Whitney U tests. Body weight was analyzed using two-way repeated-measures ANOVA with Geisser–Greenhouse correction, followed by Tukey-adjusted comparisons among time points within each group. Alpha diversity was analyzed using the Kruskal–Wallis test with false discovery rate–corrected pairwise comparisons. Beta diversity was assessed using PERMANOVA with PERMDISP. Effect sizes, 95% confidence intervals, and exact multiplicity-adjusted *p* values were reported. Individual-level raw data, complete assumption-test results, and detailed raw data are provided in Supplementary Data S1. *p* < 0.05 was considered statistically significant.

### 2.9. Statement of Reporting Guidelines

All animal procedures in this study were approved by the Ethics Committee of the Animal Care and Use Committee, Okayama University (approval no. OKU-2023464; approved on 28 April 2023) and followed the ARRIVE Guidelines 2.0 (File S1). A study protocol was prepared prior to the study, but it was not registered in a public repository.

## 3. Results

### 3.1. Body Weight Changes in Mice

**Figure 2:**
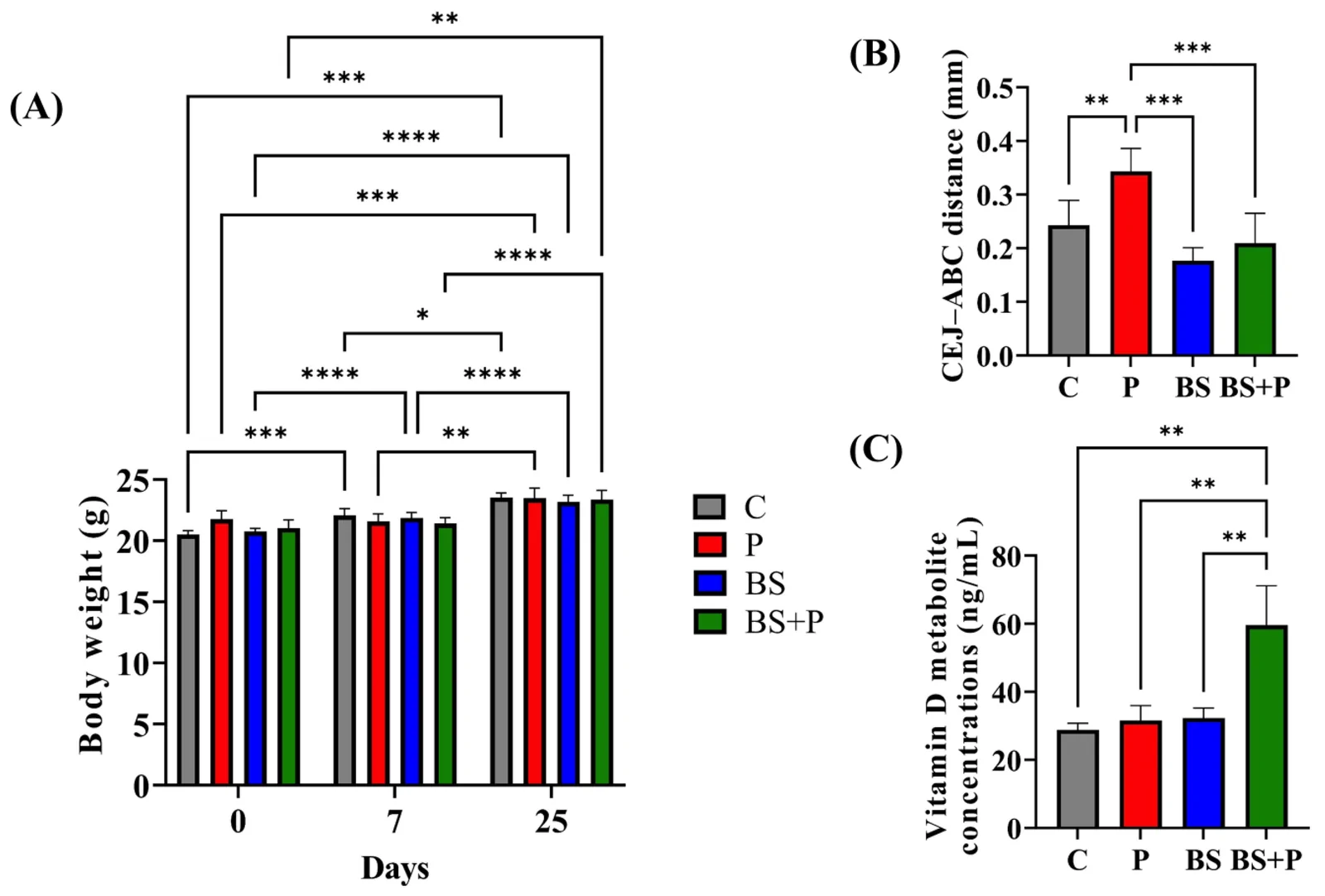
Body weight, alveolar bone loss, and ELISA-detectable serum vitamin D metabolite concentrations. (**A**) Body weight trajectories of mice in the four experimental groups on days 0, 7, and 25 (two-way repeated-measures ANOVA followed by Tukey’s test). (**B**) Alveolar bone loss assessed by cementoenamel junction-alveolar bone crest (CEJ-ABC) distance (one-way ANOVA followed by Tukey’s test). (**C**) ELISA-detectable serum vitamin D metabolite concentrations measured using a competitive vitamin D ELISA kit (Welch’s ANOVA followed by Dunnett’s T3 test). Data are presented as mean ± standard deviation (SD). C, control; P, periodontitis; BS, *Bacillus subtilis*; BS+P, *Bacillus subtilis* plus periodontitis. * *p* < 0.05; ** *p* < 0.01; *** *p* < 0.001; **** *p* < 0.0001.

Body weight changed significantly over time, with a significant group-by-time interaction (time: F (1.73, 34.52) = 278.4, *p* < 0.0001; interaction: F (5.18, 34.52) = 7.72, *p* < 0.0001), whereas the overall group effect was not significant (F (3, 20) = 0.61, *p* = 0.6191). Body weight increased from day 0 to day 7 in the C and BS groups and was higher on day 25 than on days 0 and 7 in all groups (Figure 2A). The raw data of all groups are provided in Supplementary Data S1.

### 3.2. Alveolar Bone Loss

The mean CEJ–ABC distances were 0.243 ± 0.046 mm in the C group, 0.343 ± 0.043 mm in the P group, 0.177 ± 0.024 mm in the BS group, and 0.209 ± 0.056 mm in the BS+P group. The corresponding control-normalized values were 100.0%, 141.0%, 72.7%, and 86.0%, respectively.

**Figure 3:**
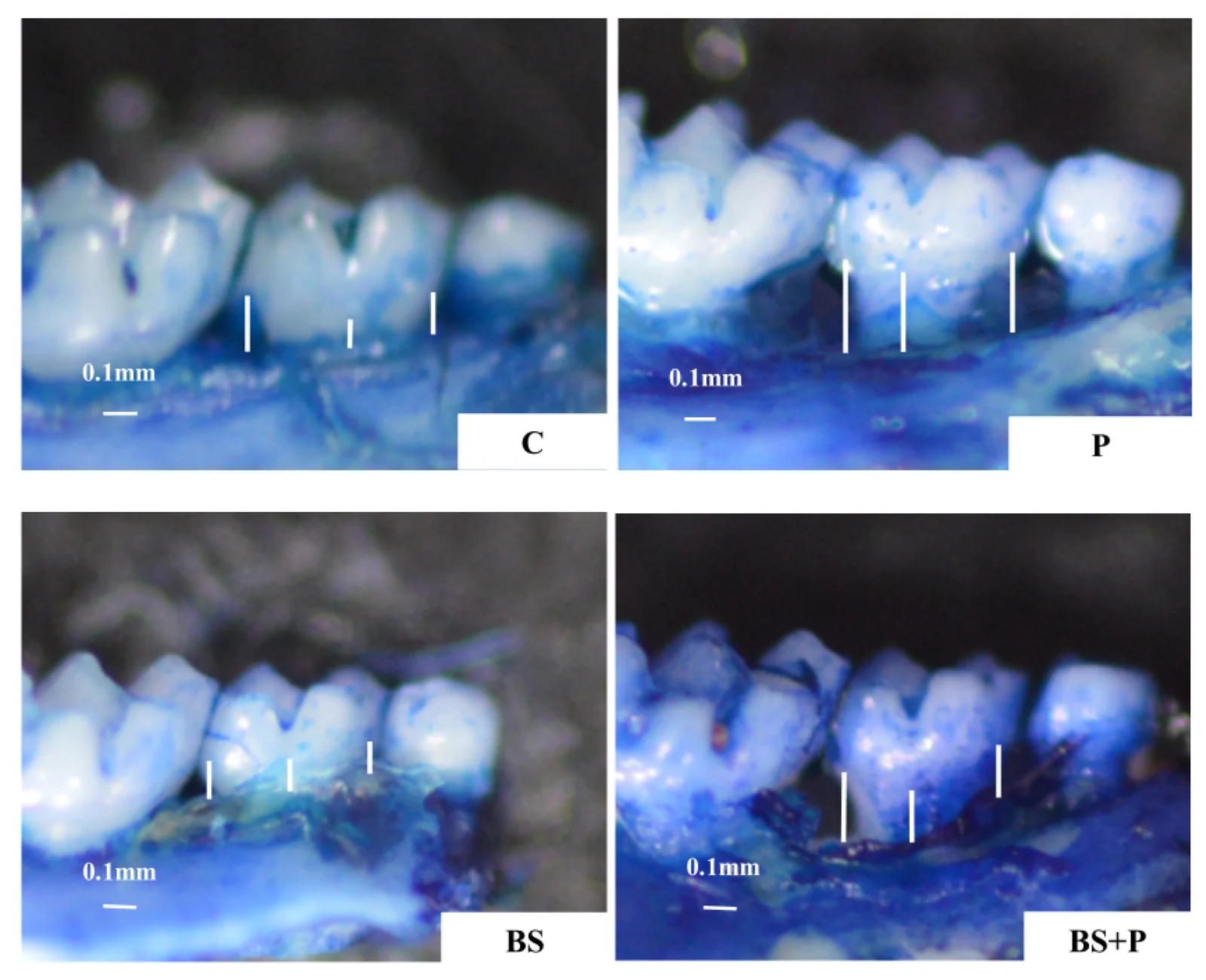
Representative stereomicroscopic images of maxillary alveolar bone in each experimental group. Maxillary bone samples were stained with methylene blue to visualize the cementoenamel junction and alveolar bone crest. The representative images are shown for the C, P, BS, and BS+P groups. The white lines indicate the three CEJ–ABC measurement sites. Scale bar = 0.1 mm. C, control; P, periodontitis; BS, *Bacillus subtilis*; BS+P, *Bacillus subtilis* plus periodontitis.

One-way ANOVA showed a significant group effect (F (3, 20) = 16.09, *p* < 0.001; ω^2^ = 0.654). The P group had a greater CEJ–ABC distance than the C group (Tukey’s test; mean difference, 0.100 mm; 95% CI, 0.029–0.171 mm; adjusted *p* = 0.0042). The BS+P group had a lower CEJ–ABC distance than the P group (BS+P minus P mean difference, −0.134 mm; 95% CI, −0.205 to −0.063 mm; Hedges’ g = −2.46, 95% CI, −3.93 to −0.94; Tukey’s adjusted *p* = 0.0002), corresponding to a 39.0% lower mean value. The BS+P and C groups did not differ significantly (Tukey’s test, adjusted *p* = 0.548; Figure 2B). The representative images are shown in Figure 3. The raw data of all groups are provided in Supplementary Data S1.

### 3.3. ELISA-Detectable Serum Vitamin D Metabolite Concentrations

ELISA-detectable serum vitamin D metabolite concentrations were 28.84 ± 1.91, 31.58 ± 4.41, 32.27 ± 2.99, and 59.61 ± 11.55 ng/mL in the C, P, BS, and BS+P groups, respectively (Figure 2C). Concentrations differed significantly among groups (Welch’s ANOVA, W (3, 10.18) = 13.28, *p* = 0.0008; Welch-adjusted ω^2^ = 0.767). The BS+P group had higher concentrations than the C, P, and BS groups (Dunnett’s T3 adjusted *p* = 0.006, 0.007, and 0.007, respectively). Compared with the P group, the BS+P group showed a mean increase of 28.03 ng/mL (95% CI, 9.53–46.53 ng/mL; Hedges’ g = 2.96, 95% CI, 1.28–4.58), corresponding to an 88.8% higher mean value. The raw data of all groups is provided in Supplementary Data S1.

### 3.4. Intestinal Morphology

**Figure 4:**
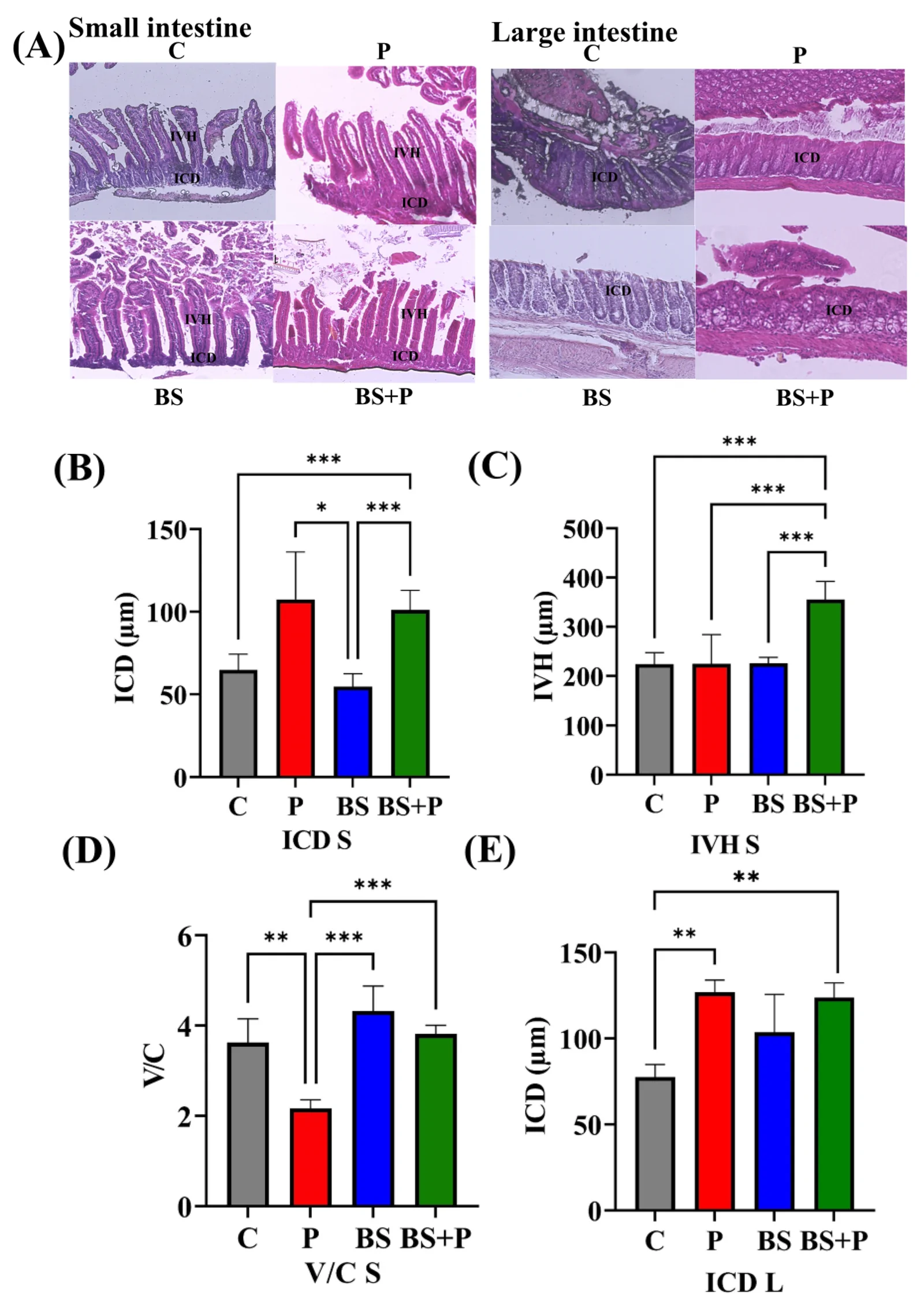
Effects of *Bacillus subtilis* supplementation on intestinal morphology in ligature-induced periodontitis mice. (**A**) Representative H&E-stained images of the small and large intestines acquired using a BZ-X810 microscope in transmitted-light bright-field mode with a ×11.1 objective lens. (**B**) Small-intestine crypt depth (ICD S). (**C**) Small-intestine villus height (IVH S). (**D**) Small-intestine villus-to-crypt ratio (V/C S). (**E**) Large-intestine crypt depth (ICD L). Data are presented as mean ± SD in panels (**B**–**E**). IVH S was analyzed using one-way ANOVA followed by Tukey’s test; ICD S and V/C S were analyzed using Welch’s ANOVA followed by Dunnett’s T3 test; and ICD L was analyzed using the Kruskal–Wallis test followed by FDR-corrected Mann–Whitney *U* tests. C, control; P, periodontitis; BS, *Bacillus subtilis*; BS+P, *Bacillus subtilis* plus periodontitis. * *p* < 0.05; ** *p* < 0.01; *** *p* < 0.001.

Analysis of intestinal morphology included all animals according to their original group allocation (*n* = 6 per group). Representative hematoxylin and eosin-stained images of the small and large intestines are shown in Figure 4A.

Small-intestine villus height (IVH S) differed significantly among groups (one-way ANOVA, F(3, 20) = 18.29, *p* = 0.00000595; ω^2^ = 0.684). IVH S was greater in the BS+P group than in the P group (mean difference, 130.70 μm; 95% CI, 70.33–191.10 μm; Hedges’ g = 2.44, 95% CI, 0.92–3.89; Tukey’s test, adjusted *p* = 0.0000353), corresponding to a 58.2% higher mean value.

Small-intestine crypt depth (ICD S) differed significantly among groups (Welch’s ANOVA, W (3, 10.65) = 22.51, *p* = 0.0000642; Welch-adjusted ω^2^ = 0.847). However, the P and BS+P groups did not differ significantly (BS+P minus P mean difference, −6.33 μm; 95% CI, −50.65 to 37.99 μm; Hedges’ g = −0.27, 95% CI, −1.31 to 0.79; Dunnett’s T3 adjusted *p* > 0.99). The BS+P group had a descriptively 5.9% lower mean value than the P group.

V/C S differed significantly among groups (Welch’s ANOVA, W (3, 10.40) = 79.89, *p* = 1.78 × 10^−7^; Welch-adjusted ω^2^ = 0.954). The ratio was higher in the BS+P group than in the P group (mean difference, 1.65; 95% CI, 1.30–2.00; Hedges’ g = 8.07, 95% CI, 4.43–11.69; Dunnett’s T3 test, adjusted *p* < 0.001), corresponding to a 75.9% higher mean value.

Large-intestine crypt depth (ICD L) also differed significantly among groups (Kruskal–Wallis test, H (3) = 14.91, *p* = 0.001898; ε^2^ = 0.595) (Figure 4). However, the P and BS+P groups did not differ significantly (BS+P minus P Hodges–Lehmann shift, −1.79 μm; 95% CI, −14.74 to 10.60 μm; rank-biserial r = −0.22, 95% CI, −0.83 to 0.50; FDR-adjusted *p* = 0.5887).

The raw data of all groups are provided in Supplementary Data S1.

### 3.5. Gut Microbiota Diversity

**Figure 5:**
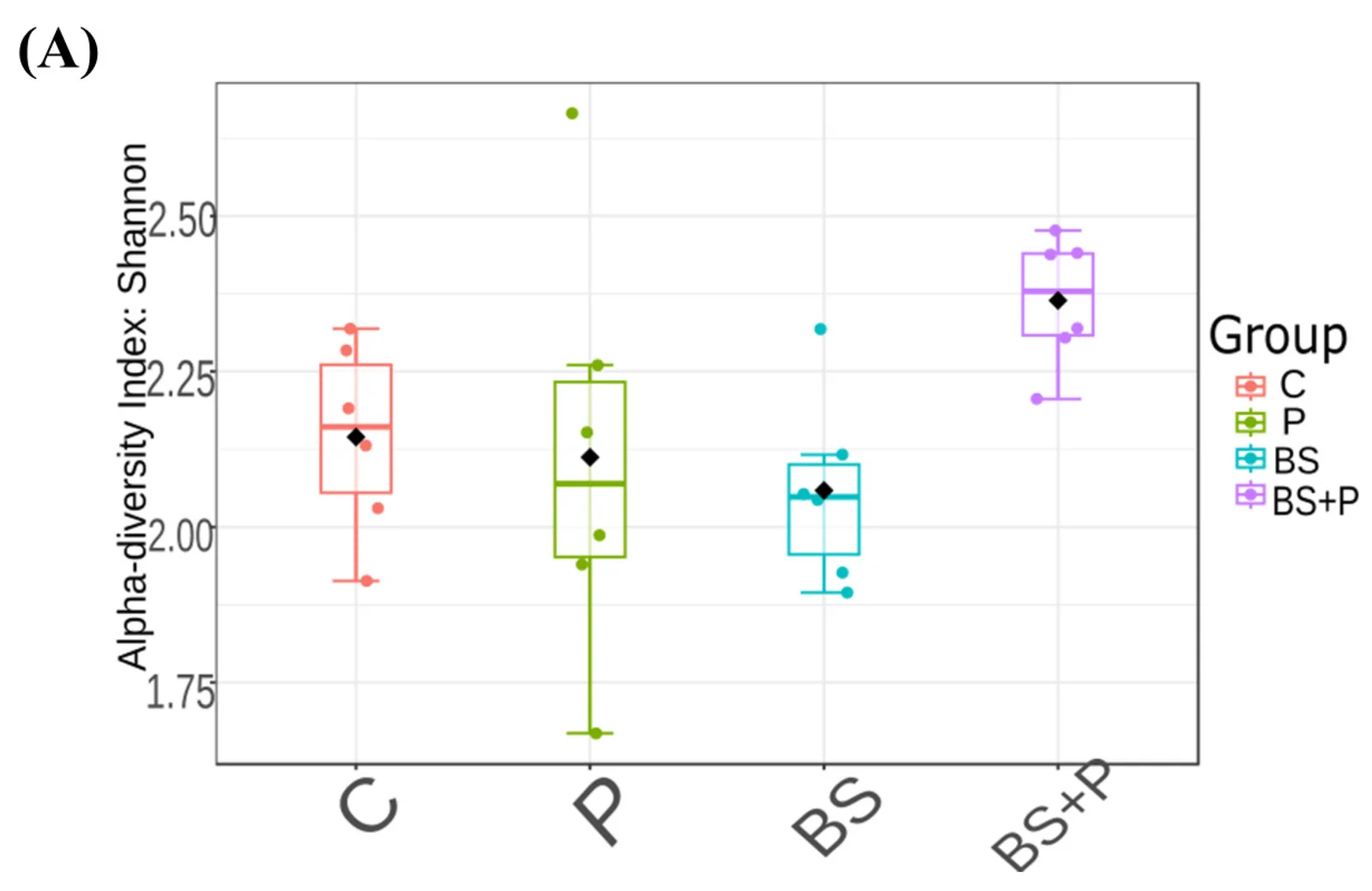

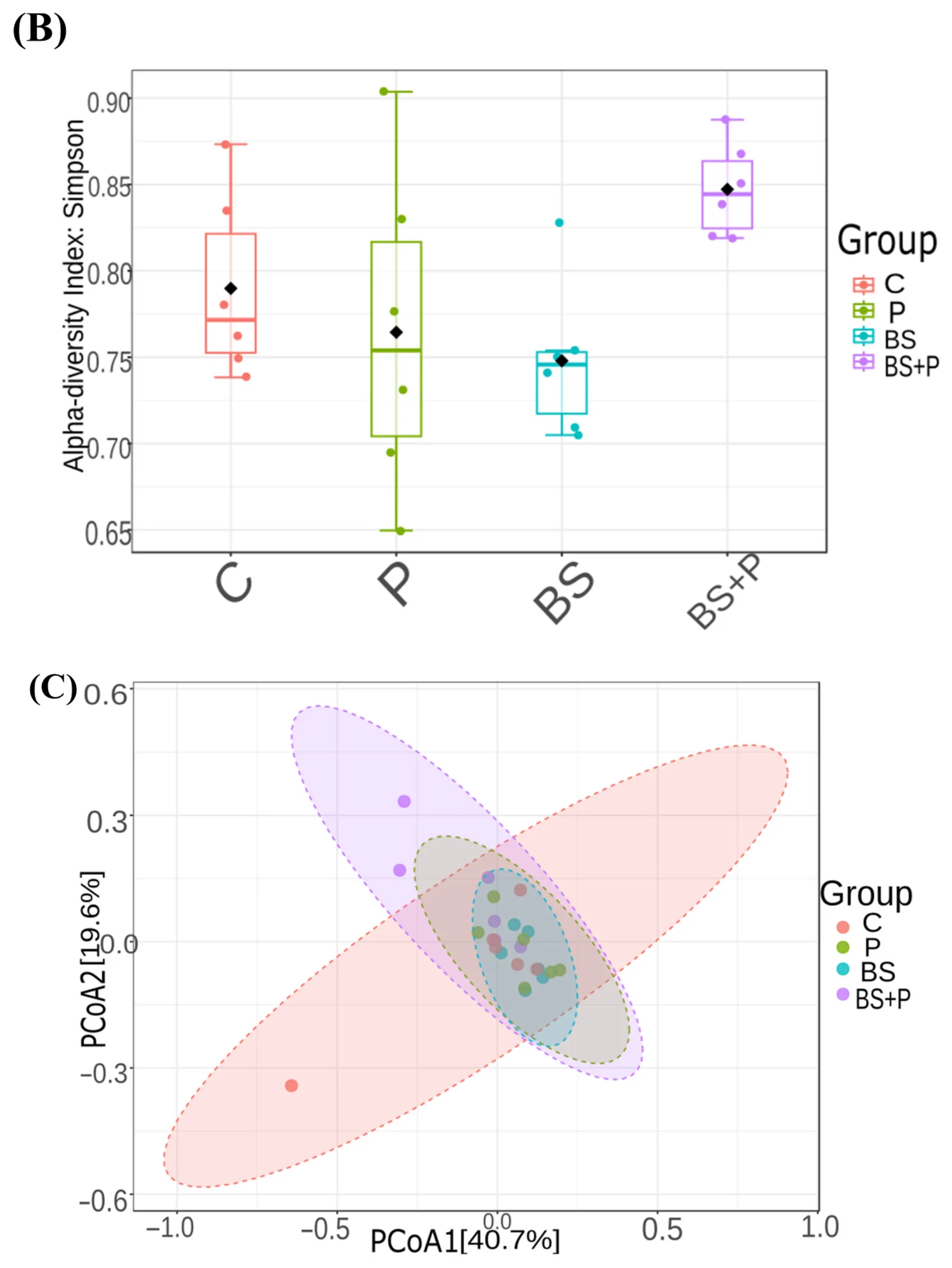
Alpha and beta diversity of day 25 fecal microbiota in the full dataset. (**A**) Shannon diversity index. (**B**) Simpson diversity index. (**C**) Principal coordinate analysis (PCoA) based on Bray–Curtis dissimilarity showing fecal microbial community composition among the four experimental groups. Black diamonds indicate group means. In the full dataset, Shannon diversity differed significantly among groups (Kruskal–Wallis, *p* = 0.038), whereas Simpson diversity showed a non-significant trend (Kruskal–Wallis test, *p* = 0.058). PERMANOVA indicated a significant difference in overall microbial community composition among groups (F = 1.765, R^2^ = 0.209, *p* = 0.019). PERMDISP did not indicate significant heterogeneity of within-group dispersion in the full dataset including C2 (F = 0.932, *p* = 0.444). C, control; P, periodontitis; BS, *Bacillus subtilis*; BS+P, *Bacillus subtilis* plus periodontitis.

In the full dataset, alpha diversity differed significantly among groups based on the Shannon index (Kruskal–Wallis test, *p* = 0.038), whereas the Simpson index did not reach statistical significance, although a trend was observed (Kruskal–Wallis test, *p* = 0.058) (Figure 5A,B). Pairwise comparisons with FDR correction showed significant differences in Shannon diversity for BS+P versus BS and BS+P versus C (Mann–Whitney *U* tests, FDR-adjusted *p* = 0.045 for both comparisons). Bray–Curtis-based beta diversity differed significantly among groups by PERMANOVA (F = 1.765, R^2^ = 0.209, *p* = 0.019), and PERMDISP did not indicate significant heterogeneity of within-group dispersion (F = 0.932, *p* = 0.444) (Figure 5C). No pairwise PERMANOVA comparison remained significant after FDR correction.

**Figure 6:**
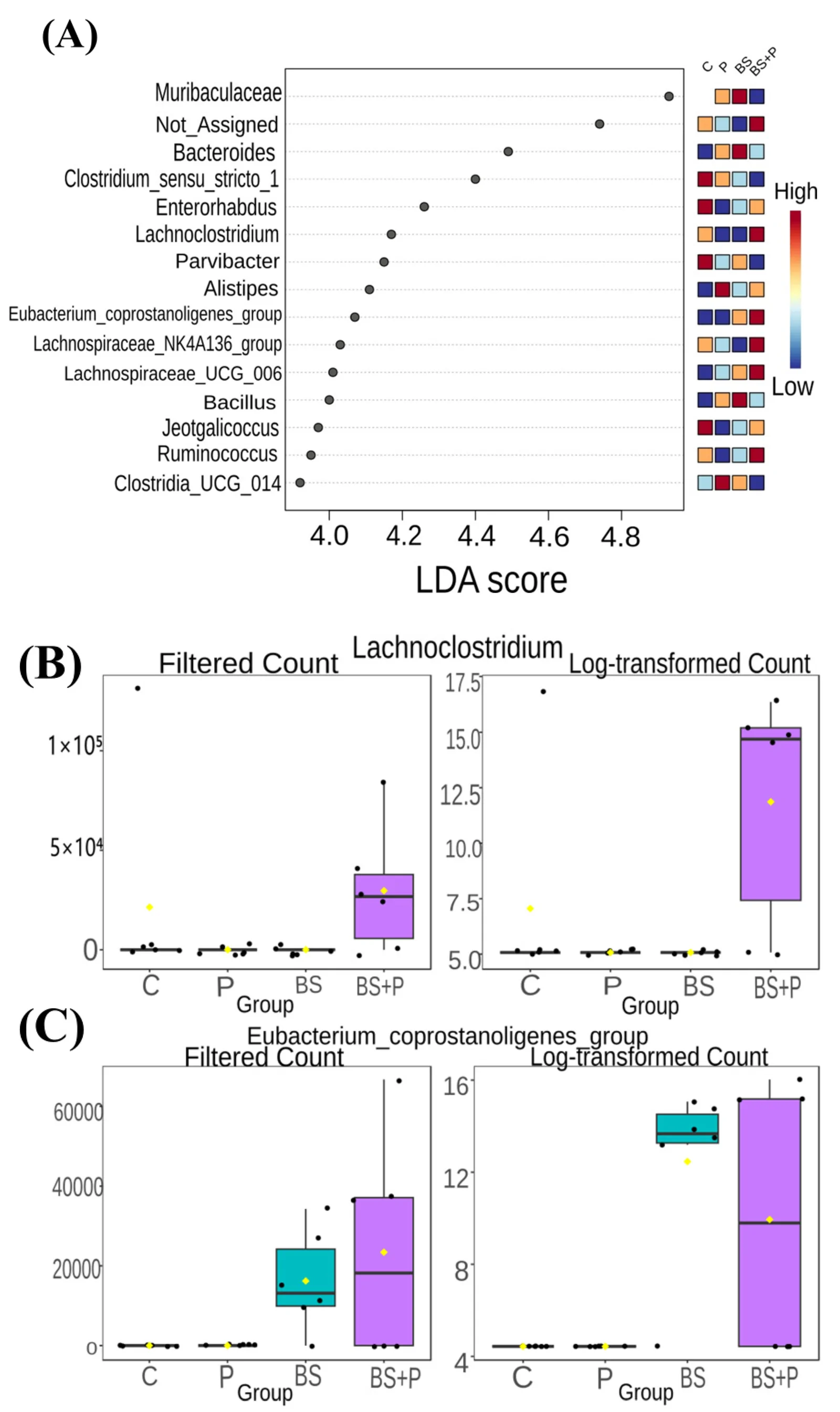
Exploratory LEfSe analysis of day 25 fecal microbiota in the full dataset. (**A**) LDA score plot showing taxa contributing to group discrimination, with an accompanying heatmap indicating relative abundance across the four experimental groups. (**B**) Filtered and log-transformed abundance patterns of *Lachnoclostridium*. (**C**) Filtered and log-transformed abundance patterns of *Eubacterium*_*coprostanoligenes*_group. Black dots represent individual samples, and yellow diamonds indicate group means. Taxon-level findings were interpreted as exploratory because of the limited sample size. C, control; P, periodontitis; BS, *Bacillus subtilis*; BS+P, *Bacillus subtilis* plus periodontitis.

**Figure 7:**
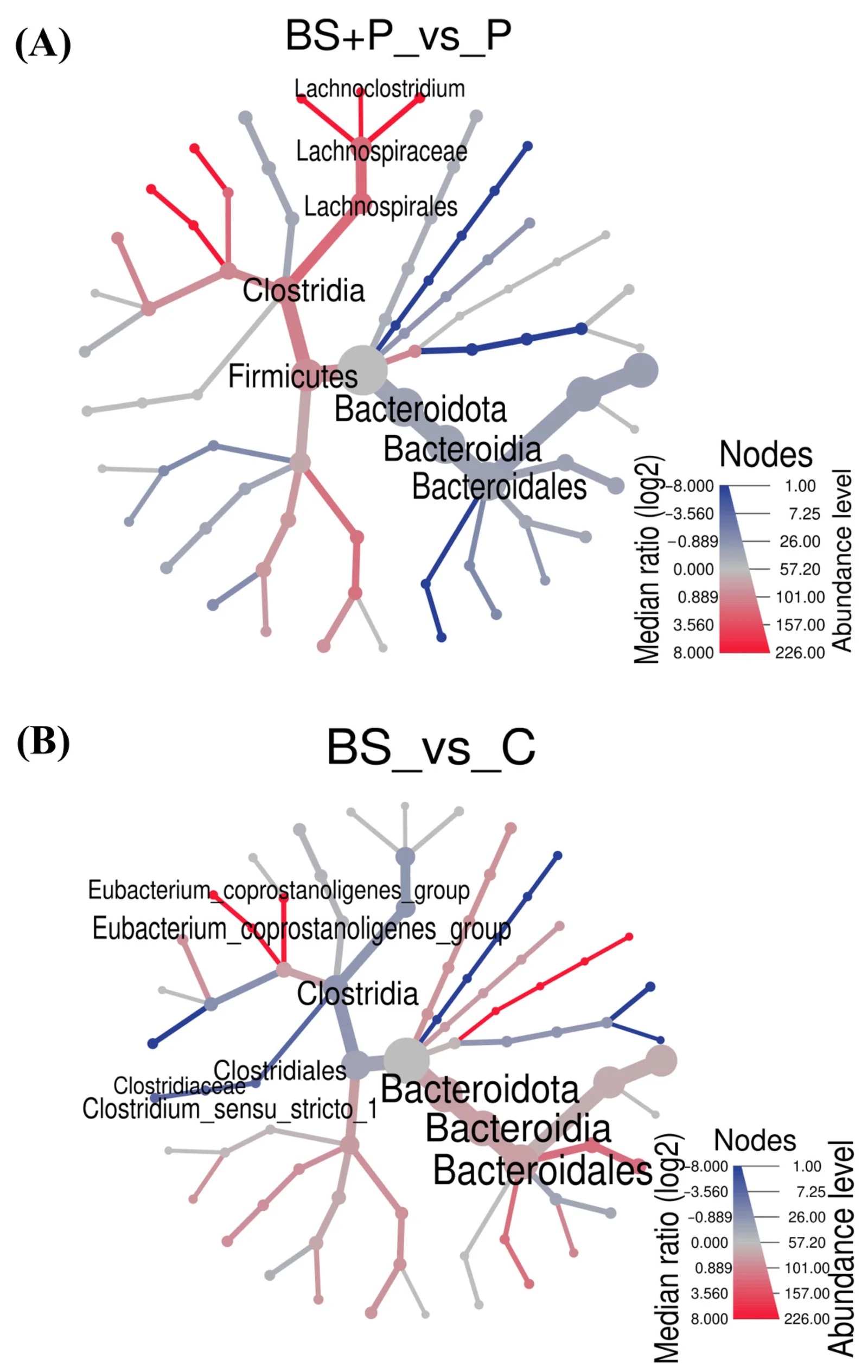
Heat-tree visualization of exploratory taxon-level abundance patterns in day 25 fecal microbiota. (**A**) Comparison between the BS+P and P groups. (**B**) Comparison between the BS and C groups. Node color represents the median abundance ratio on a log_2_ scale, and node size represents abundance level. C, control; P, periodontitis; BS, *Bacillus subtilis*; BS+P, *Bacillus subtilis* plus periodontitis.

LEfSe and heat tree analyses suggested exploratory taxon-level abundance patterns involving *Lachnospiraceae*-related and *Bacteroidota*-related taxa (Figure 6 and Figure 7).

Fecal samples were used for genus-level analysis of the gut microbiota from the four experimental groups. Rarefaction-curve analysis showed that most samples reached or approached a plateau; however, sample C2 in the C group showed markedly lower sequencing depth and did not reach saturation (Figure S1). As a sensitivity analysis, microbiota diversity was re-evaluated after excluding sample C2. After excluding C2, Shannon diversity remained significant (Kruskal–Wallis test, *p* = 0.049), Simpson diversity became significant (Kruskal–Wallis test, *p* = 0.046), and Bray–Curtis-based beta diversity remained significant by PERMANOVA (F = 2.163, R^2^ = 0.255, *p* = 0.008) (Figure S2). PERMDISP remained non-significant (F = 2.291, *p* = 0.111). Following C2 exclusion, pairwise PERMANOVA showed that only the BS+P versus BS comparison remained significant after FDR correction (pairwise PERMANOVA, FDR-adjusted *p* = 0.042).

## 4. Discussion

The present study demonstrated that oral BS administration significantly attenuated the progression of alveolar bone loss in a mouse model of ligature-induced periodontitis. The BS+P group also showed differences in intestinal morphology, gut microbial diversity, and serum vitamin D metabolite concentrations. However, these outcomes were assessed concurrently at the experimental endpoint and should be interpreted as accompanying observations rather than evidence of a confirmed mechanism.

Oral administration of BS significantly attenuated the progression of periodontitis-associated alveolar bone loss in a mouse model. This result is consistent with a previous study showing that probiotic-related interventions may attenuate experimental alveolar bone loss in association with gut microbiota-related changes [37]. In addition, administration of BS together with *B. licheniformis* produced favorable periodontal outcomes in a rat model of periodontitis [24]. More recently, a BS-derived metabolite was reported to attenuate experimental periodontitis in mice in a separate experimental setting [38]. These previous reports are consistent with our findings. However, local periodontal inflammatory cytokines, osteoclast-related markers, microbial metabolites, and signaling pathways involved in bone remodeling were not assessed in this study because of the small sample volume. Therefore, in the future, further experiments are required.

The BS+P group also showed higher serum vitamin D metabolite concentrations than in the P group. Because a comparable increase was not observed in the BS group, this finding may reflect a context-dependent response under ligature-induced periodontitis conditions rather than a general effect of BS supplementation alone. Vitamin D has been associated with periodontal health and bone metabolism [39], and previous research has shown that vitamin D-related interventions can influence bone mass and bone strength in mice [32]. Furthermore, gut microbiota, bile acids, and vitamin D were reported to be linked in another mouse model [40]. However, these pathways were not examined in this study. Thus, the higher ELISA-detectable vitamin D metabolite concentrations in the BS+P group should be regarded as a concurrent systemic observation, not a confirmed mediator of reduced CEJ–ABC distance.

Differences in small-intestine morphology were observed in the BS+P group; the most consistent changes were higher IVH and higher V/C ratio than in the P group. In a previous study, IVH and V/C were increased by BS [41], which supports our findings. In contrast, ICD in both the small and large intestine showed no significant differences between the P and BS+P groups, indicating that BS supplementation was not associated with a significant reduction in crypt depth relative to periodontitis alone. Previous studies in other models have reported associations between BS supplementation and intestinal barrier-related or inflammatory outcomes [42,43]. Recombinant BS preparations have also been examined for their effects on host immune responses in mice [44]. However, these studies do not establish the same mechanisms in the present periodontitis model. Because intestinal permeability, serum endotoxin levels, tight-junction proteins, mucin expression, and other barrier-related molecular markers were not assessed, the intestinal findings should be interpreted as histomorphometric differences rather than as evidence of restored intestinal barrier function.

The exploratory microbiota analysis showed group-level differences in Shannon diversity and Bray–Curtis-based community composition among the four experimental groups. Because sample C2 in the C group showed low sequencing depth and did not reach a rarefaction plateau, a sensitivity analysis was performed after excluding this sample. The full dataset including C2 was regarded as the primary microbiota dataset, whereas the analysis excluding C2 was used only to examine whether the low sequencing depth of this sample influenced the overall pattern. Although significant group-level differences in microbial community composition were observed in both the primary analysis and the sensitivity analysis, the small group size limited the statistical power of the microbiome analyses, particularly for pairwise comparisons and exploratory taxon-level findings. Therefore, the microbiome results should be interpreted cautiously and regarded as exploratory. LEfSe and heat tree analyses suggested exploratory taxon-level abundance patterns involving *Lachnospiraceae*-related and *Bacteroidota*-related taxa. *Lachnoclostridium* and *Eubacterium*_coprostanoligenes_group appeared to contribute to group discrimination and tended to be more abundant in BS-treated animals, especially in the BS+P group. However, microbial metabolites, functional pathways, and strain-level colonization were not measured in this study. Therefore, these taxon-level results should be considered descriptive and should be interpreted as exploratory findings rather than confirmed microbial biomarkers.

Sequences assigned to the genus *Bacillus* were not detected in one endpoint fecal sample from the BS+P group. Sensitivity analysis excluding this sample yielded similar results. Because endpoint non-detection does not necessarily indicate lack of BS exposure during the intervention, this mouse was retained in the BS+P group.

The mice received 5.0 × 10^8^ CFU of BS per day. However, the dose cannot be directly converted into a human-equivalent supplementation dose using conventional drug dose conversion methods. BS powder is commercially available and considered safe. However, the dosing regimen evaluated here has not been established for the prevention or treatment of periodontitis in humans. Thus, it is difficult to compare with previous clinical trials that used probiotics as an adjunct to non-surgical periodontal treatment [45].

This study has several limitations. First, rodent ligature-induced-periodontitis models cannot fully reproduce human periodontitis [46]. Alveolar bone loss was the primary periodontal outcome, whereas local periodontal inflammation and osteoclastogenesis were not evaluated. The absence of inflammatory cytokine measurements, RANKL/OPG assessment, TRAP staining, osteoclast counts, and histological inflammatory scores limits the mechanistic interpretation of the lower CEJ–ABC distance observed in the BS+P group. These outcomes should be included in future studies to clarify the effects of BS on local periodontal inflammation and bone resorption. Second, because no mechanistic interventions were performed, the present study cannot determine whether the observed changes in gut microbiota, intestinal morphology, or serum vitamin D metabolite concentrations contributed causally to the reduction in alveolar bone loss. Third, microbiota analysis was based on endpoint fecal samples. Temporal changes in gut microbiota, microbial metabolites, and functional pathways were not evaluated. Fourth, the present ELISA primarily detects 25-OH and/or 1α,25-(OH)_2_ vitamin D_3_ levels and may cross-react with 25-OH and/or 1α,25-(OH)_2_ vitamin D_2_. Thus, liquid chromatography and/or mass spectrometry should be considered in future studies. Fifth, because group allocation was not based on a formal randomization sequence but quasi-randomization, potential allocation bias cannot be excluded. Sixth, the external validity of this study is limited because only young male mice, a single *Bacillus subtilis* dose, one treatment duration, and one ligature-induced periodontitis model were investigated. The 18-day regimen was selected to evaluate the short-term effects of BS. Future studies should include female animals, dose–response analyses, and long-term therapeutic experiments.

## 5. Conclusions

Oral supplementation of BS attenuated the progression of ligature-induced periodontitis in a mouse model. Concurrent changes in gut microbiota, intestinal morphology, and serum vitamin D metabolite concentrations were also observed; however, these relationships remain associative, and their causal or mechanistic roles were not established.

## Data Availability

The raw 16S rRNA gene sequencing data generated in this study have been deposited in the NCBI Sequence Read Archive under BioProject accession number PRJNA1480160. Phenotypic data supporting the findings are included in the article and its Supplementary Materials. Further inquiries may be directed to the corresponding author.

## Supplementary Materials

The following supporting information can be downloaded at https://www.mdpi.com/article/10.3390/dj14080494/s1. Figure S1: Rarefaction curves for day 25 fecal microbiota samples; Figure S2: Sensitivity analysis of microbiota outcomes after excluding the low-depth C2 sample; Table S1: NCBI BioSample and SRA Run accession numbers for fecal 16S rRNA gene amplicon sequencing data; Data S1: Individual-level raw data; File S1: ARRIVE 2.0 checklist.

## Abbreviations

The following abbreviations are used in this manuscript:

ASV	Amplicon sequence variant
BS	*Bacillus subtilis*
BS+P	*Bacillus subtilis* plus periodontitis
C	Control
CEJ–ABC	Cementoenamel junction–alveolar bone crest
ELISA	Enzyme-linked immunosorbent assay
FDR	False discovery rate
H&E	Hematoxylin and eosin
ICD	Intestinal crypt depth
IVH	Intestinal villus height
LDA	Linear discriminant analysis
LEfSe	Linear discriminant analysis effect size
NCBI	National Center for Biotechnology Information
P	Periodontitis
PCoA	Principal coordinate analysis
PERMANOVA	Permutational multivariate analysis of variance
QIIME	Quantitative Insights into Microbial Ecology
SRA	Sequence Read Archive
SPF	Specific pathogen-free
V/C	Villus-height-to-crypt-depth ratio
