# Supplementary material for: Oral Supplementation of Bacillus subtilis Attenuated Alveolar Bone Loss in a Mouse Model of Ligature-Induced Periodontitis
Source: Dent J (Basel). 2026 Aug 7;14(8):494. doi: 10.3390/dj14080494 (PMC13511114; doi:10.3390/dj14080494)
Supplement: Supplementary file 1 [file dentistry-14-00494-s001.zip › File S1 ARRIVE_2.0_Checklist.pdf]

ARRIVE Guidelines 2.0 Author Checklist - Completed

Manuscript: Oral supplementation of *Bacillus subtilis* attenuated alveolar bone loss in a mouse model of ligature-induced periodontitis

Note: Section references refer to the uploaded manuscript version. If the manuscript is edited further, the section and line references should be checked again before submission.

Item	No.	Section/line number, or reason for not reporting
**The ARRIVE Essential 10**		
**Study design**	1	Sections 2.1.
**Sample size**	2	Sections 2.1.
**Inclusion and exclusion criteria**	3	Sections 2.1.
**Randomisation**	4	Sections 2.1.
**Blinding**	5	Section 2.1.
**Outcome measures**	6	Section 2.2-2.7.
**Statistical methods**	7	Section 2.8.
**Experimental animals**	8	Section 2.1.
**Experimental procedures**	9	Sections 2.1-2.7.
**Results**	10	Sections 3.1-3.5.
**The Recommended Set**		
**Abstract**	11	Lines 16-32
**Background**	12	Lines 34-79
**Objectives**	13	Lines 80-82
**Ethical statement**	14	Sections 2.9.
**Housing and husbandry**	15	Section 2.1.
**Animal care and monitoring**	16	Section 2.1.
**Interpretation/scientific implications**	17	Discussion and Conclusion.
**Generalisability/translation**	18	Discussion
**Protocol registration**	19	No public protocol registration is reported. The animal experimental protocol was reviewed and approved by the Animal Care and Use Committee of Okayama University (OKU-2023464).
**Data access**	20	Section 2.5 and Data Availability Statement.
**Declaration of interests**	21	Acknowledgments and Conflicts of Interest.

Items requiring confirmation before submission: environmental enrichment, detailed randomisation-sequence generation method, and whether group allocation was masked during data analysis were not fully specified in the manuscript. Add these details if available.
